# Demographic Consequences of Predators on Prey: Trait and Density Mediated Effects on Mosquito Larvae in Containers

**DOI:** 10.1371/journal.pone.0045785

**Published:** 2012-11-12

**Authors:** Barry W. Alto, Jolene Malicoate, Scott M. Elliott, Jacqueline Taylor

**Affiliations:** Florida Medical Entomology Laboratory, Department of Entomology and Nematology, University of Florida, Vero Beach, Florida, United States of America; Institut Pasteur, France

## Abstract

Predators may affect prey population growth and community diversity through density mediated lethal and trait mediated non-lethal effects that influence phenotypic traits of prey. We tested experimentally the roles of thinning the density of prey (lethality) in the absence of predator cues and density and trait mediated effects (lethality + intimidation) of predatory midge *Corethrella appendiculata* on competing native and invasive mosquito prey. Predator-mediated reductions in prey and density reductions in the absence of *C. appendiculata* resulted in lower percent survivorship to adulthood and estimates of the finite rate of increase (λ′) for invasive mosquito *Aedes albopictus* relative to that of controls. In most instances, thinning the density of prey in the absence, but not in the presence, of *C. appendiculata* cues resulted in lower survivorship to adulthood and λ′ for native mosquito *Aedes triseriatus* relative to that of controls. Together, these results suggested trait mediated effects of *C. appendiculata* specific to each species of mosquito prey. Release from intraspecific competition attributable to density reductions in the absence, but not in the presence, of *C. appendiculata* enhanced growth and lengthened adult lifespan relative to that of controls for *A. albopictus* but not *A. triseriatus*. These results show the importance of predator-mediated density and trait mediated effects on phenotypic traits and populations of invasive and native mosquitoes. Species-specific differences in the phenotypic responses of prey may be due, in part, to longer evolutionary history of *C. appendiculata* with *A. triseriatus* than *A. albopictus*.

## Introduction

Predators can have a major influence on prey population growth and community diversity [1]–[3]. The effect of predators on prey may largely be influenced by underlying environmental context such as the presence of other competing species [4], chemical contaminants [5]–[7], and habitat complexity [8], [9]. The most obvious effects of predators are attributable to prey capture and consumption, a direct lethal (density mediated) effect. However, the presence of predators may have non-lethal (trait mediated) effects attributable to intimidation that alter phenotypic traits of prey [10]–[12] and even extend to non-prey populations [13]. Non-lethal effects of predators may include inducing changes in behavior, development, growth, morphology and physiology [10], [14], [15]. Predator-induced changes in phenotypic traits of prey are often defensive strategies [16]–[17]. For instance, predator cues induce development of defensive spines in *Daphnia* [16], alterations in shell morphology in snails [18], heavier exoskeletons and longer caudal filaments in mayflies [19], longer caudal spines in dragonflies [20], and reduced activity and use of refugia in amphibians, insects, and other animals [14], [21]. A meta-analysis showed that the relative magnitude of non-lethal effects of predation may exceed those effects attributable to lethality [17]. Identification of non-lethal effects of predators (and measurement of their relative magnitude) is often difficult, especially when their effect is directionally similar to lethal effects of predators.

Modification of prey traits due to the presence of predators, either through lethal or non-lethal pathways, is often assumed to arise via phenotypic plasticity. However, alterations in prey traits may arise due to other processes that change phenotypes, which might include selection among individuals of different phenotypes [22]. For instance, a predator may alter the distribution of prey traits in a population via selective removal of some prey phenotypes, as demonstrated theoretically [23]–[24] and empirically [25]. Plastic responses may be reversible within an individual's life time (e.g., behavioral changes) whereas selective effects occur across generations if the traits are heritable.

Mosquitoes inhabiting water-holding containers (phytotelmata, tires, cisterns) are a tractable model system to investigate lethal and non-lethal effects of predators on phenotypic traits of prey [4], [26]. Water-filled containers are relatively simple communities that are occupied by numerous mosquito species [27]. Biotic interactions such as predation and competition are relatively common and shape mosquito communities [4] and may impact adult traits related to transmission of pathogens. For instance, intra and interspecific competition alter individual life history traits, population performance, and vector competence for arthropod-borne viruses e.g., dengue virus [28], LaCrosse virus [29], and Sindbis virus [30]–[31]. The presence of predators, as well as predator cues in the absence of capture and consumption, alters behavior and life history traits of mosquitoes including *Culex pipiens molestus* (Forskal) [32], *Aedes triseriatus* (Say) [33]–[35], and *Aedes albopictus* (Skuse) [34]–[35].

The Asian tiger mosquito *A. albopictus* (Skuse) is an invasive mosquito which has expanded is geographic range throughout much of the world in recent decades [36]. The global spread of *A. albopictus* is a public health concern since it can be a vector of numerous arthropod-borne (arbo) viruses and has been implicated as the vector responsible for recent outbreaks of dengue and chikungunya viruses. Range expansion of *A. albopictus* has been associated with declines in the abundance of the yellow fever mosquito *Aedes aegypti* (L.) in southeastern USA and in the Bermuda Islands [37], most likely attributable to the former's competitive superiority [26], [36]–[38].

Declines in the native Eastern treehole mosquito *A. triseriatus* have not been associated with establishments of invasive *A. albopictus* [39]–[40]. *Aedes albopictus* and *A. triseriatus* share similar container habitats (e.g., treeholes), some of which are also commonly occupied by predatory larvae of the mosquito *Toxorhynchites rutilus* (Coquillett) and the midge *Corethrella appendiculata* (Grabham). These *Aedes* species detect predators by water-borne and physical cues [33]–[34], [41]–[42] and potential trade-offs between competitive ability and vulnerability to predation may affect coexistence [43]–[46]. Some studies have demonstrated that *A. albopictus* is competitively superior to *A. triseriatus* in the absence of predators [47]–[51]. Laboratory and field studies have demonstrated that these species differ in their vulnerability to predation [33]–[34], [43]–[44]. Larger size of *A. triseriatus* and greater adoption than *A. albopictus* of low risk behaviors contributes to lower vulnerability of the native species to predation by *C. appendiculata* [34], [45], relationships found in other systems where prey traits contribute to resistance to predation [14], [52]. Both *A. triseriatus* and *A. albopictus* may grow to a body size relatively invulnerable to size-dependent predation by *C. appendiculata* [34], [42]. However, intra and interspecific competition may lead to food limitation which slows growth rates and lengthens exposure to predation. Empirical and theoretical studies have demonstrated that the presence of *C. appendiculata* contributes to invasion resistance and coexistence of the competing mosquito prey species [45]–[46]. However, the relative importance of thinning the density of prey and of trait mediated effects on the outcome of competition between *A. triseriatus* and *A. albopictus* has not been determined. Nonconsumptive effects of predators have been shown to reverse the outcome of competition in other systems [53]–[54] and may also have demographic consequences (e.g., growth and development) among container mosquitoes. Here we investigate these ideas in the context of competing *A. triseriatus* and *A. albopictus* and the predator *C. appendiculata*.

## Methods and Materials

### Predator, prey and experimental design

The experiment included a two-species prey community with two levels of larval treatment (intra and interspecific competition) for each prey species crossed with three levels of predation with four replicates (2×3 factorial design). Predator treatments consisted of predator absent (control), predators present and prey removal. The latter predator treatment simulated the depletion of prey by predation by removing mosquito larvae from containers according to a daily mortality schedule. The experimental containers serving as replicates consisted of cylindrical translucent plastic containers (16 cm height, 15 cm diameter) and were set up five days before the addition of mosquito larvae. Each container received 2.0 L tap water and 4.0 g *Quercus virginiana* (live oak) leaves and four days later we added 0.2 g of an equal mixture of brewer's yeast and lactalbumin. Each container was assigned a larval prey treatment and received first instar Florida-derived *A. triseriatus* and *A. albopictus* as 100∶0, 50∶50, and 0∶100, respectively. *Aedes albopictus* (generation F_4_) and *A. triseriatus* (generation > F_20_) were progeny from a colony collected as larvae from tires and treeholes in Indian River County, FL. These larval prey treatments represent intra and interspecific competition. An equal mixture of brewer's yeast and lactalbumin supplemental food (0.1 g) was added on days 6, 11, and 14 after addition of larvae. Experimental treatments were maintained at 24.2±0.5°C, 83.3±7.0% humidity and a 14∶10 hour light:dark photoperiod.

Predator present treatments consisted of the addition of four 4^th^ instar *C. appendiculata* to each experimental container. Larvae of *C. appendiculata* were obtained from a Florida colony established from 200–300 individuals collected from water-filled containers (treeholes, tires). Rearing of *C. appendiculata* used methods described in [55]. Mosquito larvae in predator present treatments were exposed to non-lethal predator cues (trait mediated effects) as well as capture and consumption (density mediated effects) by *C. appendiculata*. Predator absent (control) treatments lacked the addition of *C. appendiculata* and so mosquito larvae only experienced intra and interspecific competition. We simulated the thinning effect of predation according to daily mortality schedules using a prey removal treatment. Thus, we exclude trait mediated effects attributable to the presence of *C. appendiculata* (e.g., changes in prey behavior). Every day the entire contents (water + larvae) of all experimental containers were emptied into enamel pans, subjected to treatment manipulation, and then returned to their original containers. Treatment manipulations included counting prey mosquitoes, removal of prey (i.e., in removal treatments), and maintaining *C. appendiculata* density the same from day to day (i.e., in the predator present treatments). For predator present treatments we enumerated mosquito larvae and removed and replaced dead *C. appendiculata* or pupae with 4^th^ instar *C. appendiculata*. Enumerating mosquito larvae was the basis for creating daily mortality schedules. No attempt was made to identify the species of mosquitoes before emergence to adulthood. Each prey removal treatment replicate was paired with a predator present treatment replicate for the duration of the experiment. Every day equal numbers of larvae were removed from prey removal treatments based on the daily estimated mortality from paired predator treatments. A numbered grid consisting of 4×4 cm cells was drawn on the bottom of enamel pans used for holding the removal treatment contents. A randomly chosen number representing one of the cells was used to identify a cell to remove larvae. All larvae were removed from one cell before another random number was identified and larvae were removed from the second chosen cell. This process was repeated until the desired numbers of larvae were removed from each prey removal treatment replicate. The methodology used for removal treatments minimize the possibility for selective removal of one mosquito species over the other. Thus, we isolated the effects of density manipulation but did not account for species-specific prey selection in treatments where both species were present. The prey removal treatment simulated the thinning effect of predation by *C. appendiculata* (lethality) without associated trait mediated effects (e.g., prey behavioral modification) that may occur in the presence of *C. appendiculata*. The predator present treatment includes both density and trait mediated effects of *C. appendiculata* on mosquito prey (lethality + intimidation). In most natural situations, both density and trait mediated effects of predation occur simultaneously which was our reasoning for use of this latter treatment. Thus, we did not assess perception of predation risk independently from density-mediated effects.

### Data measurements

Mosquito pupae from experimental containers were collected daily and transferred to plastic vials for adult emergence. Because pupae were transferred from the experimental containers to vials, mosquitoes were exposed to predators for only part of the time during their pupal stage. Newly emerged adult mosquitoes were scored by species and sex and the females were transferred daily to 0.5 L paperboard cages (1–5 females/cage) with mesh screening with continuous access to water-soaked cotton. Adult females were monitored for survivorship daily between 0900 and 1500 hours during the light period of the 24 hour daily cycle. Dead adults were recorded and stored at −20°C. We gauged treatment effects on mosquito prey by measuring percent survivorship to adulthood from the initial cohort of larvae, development time to adulthood, female dry weight and adult female lifespan. An estimated finite rate of increase (λ′) was calculated for each treatment replicate:


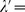
 *exp* 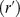  


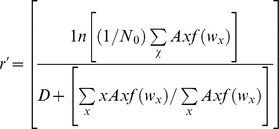
where *N_o_* is the original number of females in a cohort (assumed to be 50%), *A_x_* is the number of females emerging to adulthood on day *x*, *w_x_* is the mean adult female size on day *x*, and *f*(*w_x_* ) describes the relationship between female size and the number of eggs produced. Masses of adult *A. triseriatus* and *A. albopictus* were determined by measuring dry weights (dried at 80°C for >48 h) on an electrobalance. *D* is the number of days from adult female emergence to oviposition. *D* is set at 12 days for *A. triseriatus* and 14 days for *A. albopictus* [47], [56]. We used the following fecundity-size relationships [*f*(*w_x_* )]:

*A. triseriatus* 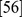


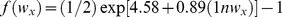


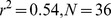



*A. albopictus* 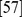


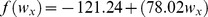


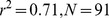



### Statistical analyses

Treatment effects on mosquito responses were analyzed separately for *A. triseriatus* and *A. albopictus*. Treatment effects on mosquito survivorship, development time (female, male), and weight of adult females were analyzed using multivariate analysis of variance (MANOVA). Standardized canonical coefficients were used to determine the relative contribution of each response variable to significant MANOVA effects. Treatment effects on mosquito λ′ were analyzed using analysis of variance. When significant effects were detected, we used pairwise contrasts of means adjusting α = 0.05 for multiple comparisons (Tukey-Kramer adjustment for multiple comparisons, PROC GLM, SAS 9.22). Treatment effects on survival probability of adults, using lifespan of adult females, were compared using non-parametric survival analysis (PROC LIFETEST, SAS 9.22). Follow-up procedures used logrank test statistics to compare pairwise estimates of the survival probability functions adjusting for multiple comparisons using the Sidak method [58]. Adult lifespan was measured for 334 and 193 female adults for *A. albopictus* and *A. triseriatus*, respectively.

## Results

**Figure 1:**
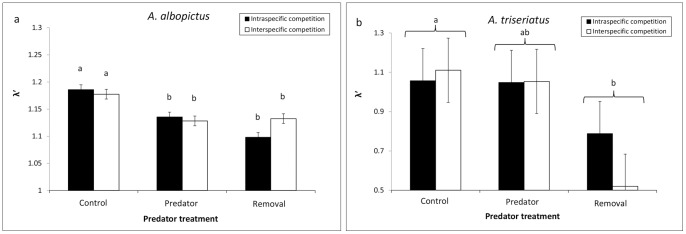
Estimated finite rate of increase (λ'). λ′ of a) *A. albopictus* and b) *A. triseriatus* for a significant interaction between predation and intra and interspecific competition for *A. albopictus* and a significant effect of predation treatment for *A. triseriatus*. Treatments followed by different letters are significantly different (*P*≤0.05) from one another. Brackets denote significant differences between treatments that include the cumulative effect of two mean values.

Analysis of variance demonstrated significant effects of the predator treatment on λ′ of both *A. albopictus* and *A. triseriatus*. Also, there was an interaction between the predator and intra and interspecific treatments on λ′ of *A. albopictus* (Table 1). *Aedes albopictus* in the control treatments had significantly higher λ′ than in predator present and prey removal treatments (Figure 1a). Despite indication of a significant interaction, after correcting for multiple comparisons, the intra and interspecific treatments had similar effects on λ′ of *A. albopictus* (Figure 1a). *Aedes triseriatus* λ′ in control treatments was significantly higher than in prey removal but not in predator present treatments (Figure 1b). Differences between the predator present and prey removal treatments were not signficant for *A. triseriatus* but trends (*P* = 0.06) showed higher λ′ in the predator present than the removal treatments (Figure 1b).

**Table 1:** Analysis of variance results for λ′′ of *A. triseriatus* and *A. albopictus* in response to treatment effects of predation, intra and interspecific competition, and their interaction.

	*A. triseriatus*			*A. albopictus*		
Source	d.f.	*F*	*P*	d.f.	*F*	*P*
Predation (P)	2	4.28	**0.0301**	2	29.87	**<0.0001**
Competition (C)	1	0.27	0.6075	1	0.72	0.4080
P × C	2	0.56	0.5798	2	3.74	**0.0440**
Error	18			18		

Significant effects are shown in boldface.

**Figure 2:**
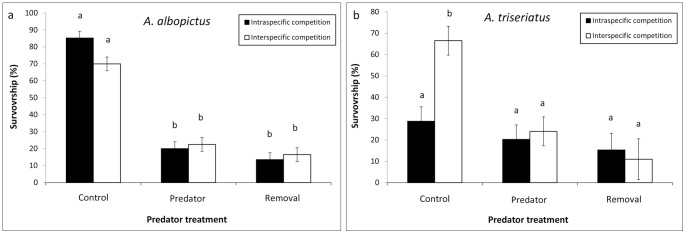
Survivorship to adulthood. Survivorship of a) *A. albopictus* and b) *A. triseriatus* for predation and intra and interspecific competition treatments. Treatments followed by different letters are significantly different (*P*≤0.05) from one another.

Multivariate analysis of variance showed a significant predator treatment effect on *A. albopictus* (Table 2). The magnitudes of the standardized canonical coefficients indicated that differences in percent survivorship to adulthood followed development time of females contributed the most to the significant predator effect (Table 2). Differences in development times of males and dry masses of females contributed to a lesser extent to the significant predator effect (Table 2). *Aedes albopictus* in the control treatments had significantly higher survivorship to adulthood than in the predator present and prey removal treatments (Figure 2a). Development time of females, but not males, were significantly longer in the control treatments than in the predator present and prey removal treatments (mean±S.E.; control, 10.15±0.08; predator present, 9.20±0.08; prey removal, 9.28±0.08). Pairwise contrasts of means for the predator treatments showed that adults were significantly larger in the prey removal treatment relative to the control and predator present treatments (mean±S.E.; control, 0.382±0.005; predator present, 0.399±0.005; removal, 0.425±0.005). Masses of *A. albopictus* were not significantly different between the control and predator present treatments.

**Table 2:** Multivariate analysis of variance on survivorship, development (female, male), and weight of adult females in response to predation and intra and interspecific treatments.

				Standardized canonical coefficients (SCCs)			
Source	Pillai's Trace	d.f	*P*	Survivorship	Female development	Male development	Female weight
*A. triseriatus*							
Predation (P)	0.96	8, 26	**0.0163**	2.61	0.45	−0.96	−1.34
Competition (C)	0.77	4, 12	**0.0007**	−2.39	−0.32	1.18	1.80
P × C	1.04	8, 26	**0.0067**	−2.38	0.05	1.40	1.58
*A. albopictus*							
Predation (P)	1.41	8, 32	**<0.0001**	3.25	0.85	−0.16	−0.18
Competition (C)	0.30	4, 15	0.2211	−1.41	2.01	−0.41	0.35
P × C	0.63	8, 32	0.1032	1.39	−1.12	0.85	−0.91

Significant effects are shown in boldface.

Multivariate analysis of variance showed significant predator treatment, intra and interspecific treatment and interaction effects on *A. triseriatus* (Table 2). Standardized canonical coefficients indicated that differences in percent survivorship to adulthood followed by dry masses of females and development time of males contributed the most to the significant interaction treatment effect. Differences in development time of females had only a minor contribution to the interaction effect (Table 2). The interspecific competition treatments for *A. triseriatus* had significantly greater percent survivorship to adulthood in the control than other treatments (Figure 2b). Pairwise comparisons of the means of predator x intra and interspecific competition treatments for development times and dry masses of *A. triseriatus* were not significant.

**Figure 3:**
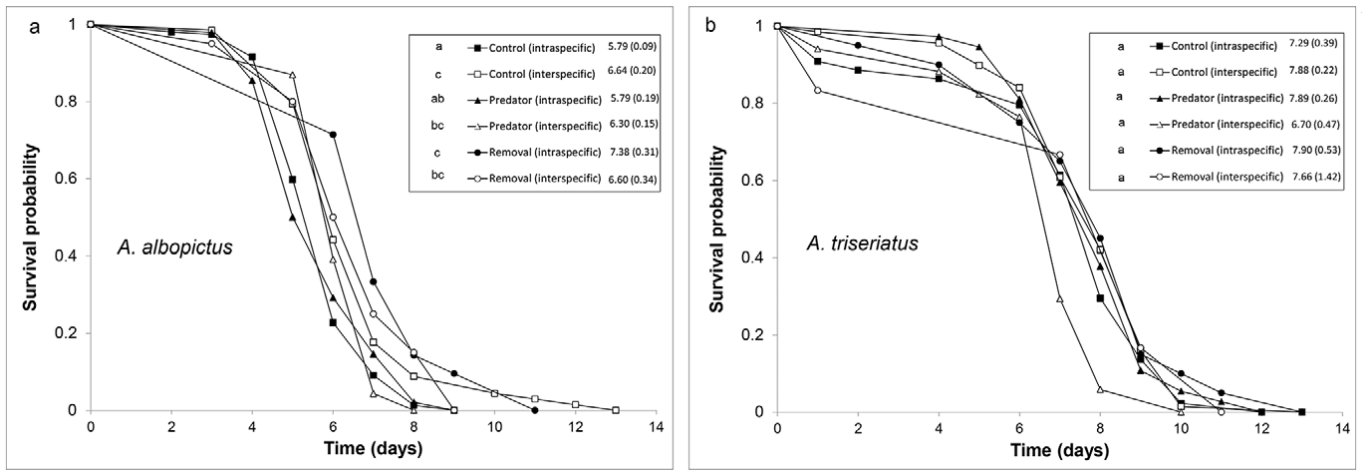
Survival of adult female mosquitoes. Survival (proportion) probability of a) *A. albopictus* and b) *A. triseriatus* adult females. For *Ae. albopictus* there was a significant interaction between predator and intra and interspecific competition treatments. Treatments followed by different letters are significantly different (*P*≤0.05) from one another. Numbers in the figure legends indicate mean (± standard error) lifespans in days.

Non-parametric survival analyses demonstrated significant effects of the predator treatment, intra and interspecific treatment, and the interaction on survivor function estimates of *A. albopictus* females (Table 3). The survivor function is the probability that mosquitoes survive until time *t*. Comparison of survival distributions showed significantly steeper declines in survivor function estimates of adults from the control (intraspecific) treatments than all other treatments. However, the control (intraspecific) and predator (intraspecific) treatments were not significantly different from one another (Figure 3a). Pairwise contrasts of survivor functions for predator x intra and interspecific competition treatments showed steeper declines in survivor function estimates of adults from the predator (intraspecific) treatments than the prey removal (intraspecific) and control (interspecific) treatments (Figure 3a). Survival function estimates of *A. triseriatus* were not affected by the treatments (Table 3, Figure 3b).

**Table 3:** Non-parametric analysis for survival probability of adult *A. triseriatus* and *A. albopictus* females in response to treatment effects of predation, intra and interspecific competition, and their interaction.

	*A. triseriatus*			*A. albopictus*		
	d.f.	?^2^	*P*	d.f.	?^2^	*P*
Source						
Predation (P)	2	2.9278	0.2313	2	14.2592	**0.0008**
Competition (C)	1	0.0210	0.8847	1	11.8322	**0.0006**
P × C	5	9.115	0.1047	5	34.2433	**<0.0001**

Significant effects are shown in boldface.

## Discussion

To explore the relative roles of density mediated versus density and trait mediated effects on competing mosquitoes, we exposed competing *A. triseriatus* and *A. albopictus* to the thinning effect of predation without predators (lethality) as well as treatments where the predator *C. appendiculata* were present (lethality + intimidation). Previous studies have demonstrated that interspecific interactions between *A. triseriatus* and *A. albopictus* may be altered by the presence of *C. appendiculata*. Specifically, in the presence of *C. appendiculata*, the competitive advantage that *A. albopictus* has over *A. triseriatus* is diminished [43], [45]–[46]. In contrast, a meta-analysis showed competitive equivalence and no context dependence on competitive outcome between these two species [59]. Given that *A. triseriatus* is less vulnerable to predation by *C. appendiculata* than *A. albopictus*, we expected that *A. triseriatus* in interspecific treatments would perform worse in prey removal than in predator present treatments. However, neither the prey removal treatment nor the predator present treatment altered the outcome of interspecific interactions between *A. triseriatus* and *A. albopictus*, providing equivocal support for predator-mediated changes in competitive outcome. Interspecific competition is resource-dependent [60]–[61] and so nutrients may not have been sufficiently limiting to detect interspecific differences between *A. triseriatus* and *A. albopictus*. These results support a meta-analysis showing equivalent effects of inter- and intraspecific competition of *A. triseriatus* and *A. albopictus* [59].

Survivorship and λ′ were clearly altered by the presence of *C. appendiculata* and the thinning effect of prey in the absence of predators, but effects differed between prey species. The predator present and prey removal treatments reduced λ′ of *A. albopictus* relative to control treatments, suggesting that *C. appendiculata* effects were largely attributable to density and not trait mediated effects. In contrast, λ′ of *A. triseriatus* were similar in the presence of *C. appendiculata* and controls lacking predators. Therefore population growth of *A. albopictus* was reduced in the presence of *C. appendiculata* whereas population growth of *A. triseriatus* did not substantially differ from the controls. Greater effects of *C. appendiculata* on λ′ of *A. albopictus* may have been due to recent evolutionary contact with this predator whereas *A. triseriatus* has had longer evolutionary history with *C. appendiculata*. Variation in survival to adulthood contributes strongly to changes in λ′. For *A. albopictus*, the presence of *C. appendiculata* reduced survivorship by approximately 77% for intraspecific and 68% for interspecific treatments relative to the controls. A similar comparison for *A. triseriatus* showed a lower reduction of approximately 30% for intraspecific and 64% for interspecific treatments in percent survivorship. Our findings were consistent with other studies showing that *A. triseriatus* is less susceptible to capture and consumption by *C. appendiculata* than *A. albopictus* [34]. Regardless, estimates of λ′ were always greater than 1 for *A. albopictus* for all treatment groups, whereas some estimates of λ′ for *A. triseriatus* were less than 1 indicating that populations were declining.

Survival of *A. triseriatus* was similar in all treatments except interspecific controls which were substantially higher than other treatment groups. These results differ from those of studies showing that in the absence of predators, interspecific competition with *A. albopictus* has a greater impact than intraspecific competition on *A. triseriatus* [47]–[51] but support a meta-analysis suggesting competitive equivalence [59]. The cause of this difference from other studies is unknown, but release from competition may have occurred due to the addition of supplemental larval food during the experiment. Also, differences in the timing of development and emergence between the species may have been contributing factors. Specifically, male and female *A. albopictus* emerged as adults within ∼8–10 days whereas *A. triseriatus* required ∼11–15 days. Therefore, *A. triseriatus* in interspecific treatments experienced release from competition after *A. albopictus* developed into pupae (a non-feeding stage) which would have occurred before the addition of more larval food resources 11 and 14 days after larvae were added to containers. Additionally, cohorts were synchronized and so there were few or no stragglers as larvae, another sign of release from competition.

Capture and consumption of prey by predators may decrease development time of prey by releasing survivors from competition [44], [59], [62]–[63] or through selective consumption of slow developing prey [64]. Shorter development times were observed for *A. albopictus* in the predator present and prey removal treatments than in the controls. Also, survivorship to adulthood was approximately four times higher in controls than in the former two treatments. Together these effects indicate that competition was occurring because thinning of mosquito prey by *C. appendiculata* or prey removal accelerated development. Similar developmental times to metamorphosis of *A. albopictus* between the predator and removal treatments suggest that *C. appendiculata* did not reduce activity and foraging of prey [65], prolonging development. These results are consistent with Griswold and Lounibos [44] showing decreased development time of *A. albopictus*, but not *A. triseriatus*, in the presence of *C. appendiculata. Corethrella appendiculata* is a size-limited predator and so the lack of predator effects on *A. albopictus* males may be in part due to rapid development and the achievement of larger sizes less vulnerable to *C. appendiculata*.

Prey exposed to predation may experience enhanced growth among survivors [44], [59], [62]–[63] from competitive release or size-selection favoring larger individuals among survivors [64], [66]. This effect is especially pronounced among size-limited predators where prey may reach a size refuge [24]. We observed enhanced growth of *A. albopictus* in removal treatments simulating daily thinning of prey by *C. appendiculata* but not in the predator present treatments. The lack of enhanced growth of *A. albopictus* in the predator present treatments suggests that nonconsumptive effects of *C. appendiculata* oppose thinning effects of predation for mosquito growth. Lounibos et al. [67] observed smaller sized *A. triseriatus* adult females from tires with *T. rutilus* compared to tires where *T. rutilus* was absent and suggested a plausible mechanism; reduced foraging activity leading to decreased size at metamorphosis. Studies have since established that *A. triseriatus*, and to a lesser extent *A. albopictus*, do reduce activity in the presence of *T. rutilus* and *C. appendiculata* [33]–[34], [65], [35]. In the current study, if *A. albopictus* reduced activity and foraging in presence of *C. appendiculata* we would expect associated lengthening of development time. However, we observed the exact opposite result. The size and timing of metamorphosis for organisms with complex life cycles is tightly associated with variation in environment. Field studies on spatial and temporal patterns of phenotypic variation in size and timing of metamorphosis of the mayfly *Baetis bicaudatus* showed that sizes were smallest in streams with predatory fish and the sizes decreased with predatory stonefly density [68]. Predator-mediated reductions in sizes of mayflies were associated with accelerated development. These effects were independent of resources, competitor densities, and other physical and chemical variables. The authors proposed that variation in size and timing of metamorphosis represented adaptive developmental plasticity. Although definitive evidence is lacking, the results of this study are consistent with those of other studies showing adaptive developmental plasticity in response to variation in risk of predation for *A. albopictus*.

Predator intimidation in the absence of prey culling may have strong influence over phenotypic traits of prey [10], [17] including alterations in lifespan [35]. Thinning of prey by removal and the presence of *C. appendiculata* produced opposite effects on survivor function estimates of adults of *A. albopictus* from intraspecific treatments. In the absence of *C. appendiculata*, thinning of prey increased survival probability (longest lifespans) through competitive release whereas thinning of prey in the presence of *C. appendiculata* reduced survival probability (shortest lifespans). Thus, the impact of intimidation by *C. appendiculata* comes at an energetic cost to prey realized by reductions in growth and survival probability of adults. This is one of a few studies that have identified increased probability of death (life-shortening) effects on mosquitoes due to trait mediated effects of predators [35]. However, our experiment did not independently assess trait and density mediated effects of *C. appendiculata*. That is, no treatments included only predator cues or predator cues in combination with simulated daily reductions in density via prey removal. Rather, our treatments were limited to lethality, lethality + intimidation, and controls.

Biological control agents targeting the immature stages of mosquitoes (e.g., predatory fish, crustaceans and insects; parasitic fungi and nematodes) are assumed to reduce risk of disease transmission through reductions in the density of adult vector mosquitoes. Observed predator effects on *A. albopictus* suggest an unanticipated benefit of biological control by predators i.e., the impact of intimidation resulting in reductions in growth, and associated fecundity, and daily survival probability of adults. These effects have yet to be observed under field conditions and so should be interpreted with caution. Vector-borne diseases are sensitive to altered vector lifespan, especially when the extrinsic incubation period of the pathogen approaches life expectancy. Control interventions that reduce the daily survival probability of adult mosquitoes via insecticides or the life-shortening bacteria *Wolbachia* are predicted to reduce transmission. In the current experiment, predator effects on *A. albopictus* survival probability of adults were complex as they also depended on interspecific interactions with *A. triseriatus*. Specifically, the life-shortening effects of *C. appendiculata* were not observed in *A. albopictus* adults reared with *A. triseriatus* during the immature stages. Along the same lines, adult life-shortening effects attributable to high larval densities during the immature stages (controls) were observed for *A. albopictus* in the intra but not interspecific treatments with *A. triseriatus*. Interspecific interactions with *A. triseriatus*, but not conspecifics, confers a lifespan advantage for *A. albopictus*.

The results of this study highlight the need to improve our understanding of how mosquitoes respond to predators that are naturally present or intentionally released in the environment as a means of controlling mosquito pests and vectors of disease agents. Different pathways contribute to effects of predators on phenotypic traits and population growth of mosquitoes [12]. The current study was a necessary starting point to first establish whether the thinning effect of simulated predation were commensurate with consumptive and nonconsumptive effects of *C. appendiculata*. In some instances, there were nonconsumptive (trait mediated) effects of predation. In this context, studies should aim to make use of treatment manipulations that enable further dissection of density reduction, predation cues, and selective predation on phenotypic traits of mosquito prey and population growth.
